# Supplementary material for: Water-resistant redox-active metal–organic framework
Source: Nat Commun. 2025 Dec 1;16:10798. doi: 10.1038/s41467-025-65849-y (PMC12669745; doi:10.1038/s41467-025-65849-y)
Supplement: Supplementary file 1 — Supplementary Information [file 41467_2025_65849_MOESM1_ESM.pdf]

Supplementary Information

Water-Resistant Redox-Active Metal–Organic Framework

Ryota Akai[1], Showa Kitajima[1], Kohei Okubo[1], Nobuyuki Serizawa[2], Hirotomo Nishihara[1,3,] Hitoshi Kasai[1], Kouki Oka[1,4,5]*

[1]Institute of Multidisciplinary Research for Advanced Materials, Tohoku University, 2-1-1 Katahira, Aoba-ku, Sendai, Miyagi 980-8577, Japan. [2]Department of Applied Chemistry, Faculty of Science and Technology, Keio University, 3-14-1 Hiyoshi, Kohoku-ku, Yokohama, Kanagawa 223-8522, Japan. [3]Advanced Institute for Materials Research (WPI-AIMR), Tohoku University, 2-1-1 Katahira, Aoba-ku, Sendai, Miyagi 980-8577, Japan. [4]Carbon Recycling Energy Research Center, Ibaraki University, 4-12-1 Nakanarusawa, Hitachi, Ibaraki 316-8511, Japan. [5]Deuterium Science Research Unit, Center for the Promotion of Interdisciplinary Education and Research, Kyoto University, Yoshida, Sakyo-ku, Kyoto 606-8501, Japan. (E-mail: oka@tohoku.ac.jp)

Experimental Section

1.1 Materials.

Acetic acid and ammonium hydrogen carbonate were purchased from Nacalai Tesque Inc. (Japan), 2,5-dihydroxyterephthalic acid was purchased from Tokyo Chemical Industry Co., Ltd. (Japan), Super P Conductive Carbon Black (Super P) was purchased from MTI Co., and single-walled carbon nanotubes (SWNTs) were purchased from Meijo Nano Carbon Co., Ltd. (Japan). Other chemicals were purchased from FUJIFILM Wako Pure Chemical Corporation (Japan).

1.2 Measurements

Microwave-assisted synthesis was performed using the Initiator+ (Biotage, Sweden). Centrifugation was performed using the Centrifuge H-19α (Kokusan, Japan). Field emission scanning electron microscopy (FE-SEM) was performed using a JSM-7800F (JEOL Ltd., Japan); the measurements were carried out at an acceleration voltage of 5 keV and a working distance of 3 mm in USD mode. Powder X-ray diffraction (PXRD) was performed with a SmartLab 3G (Rigaku, Japan) using graphite monochromatized Cu-Kα radiation (λ = 1.5418 Å) at 25°C. Fourier-transform infrared (FT-IR) spectra were recorded on the IRSpirit spectrophotometer (Shimadzu, Japan). Thermogravimetric analysis was performed using the DTG-60A (Shimadzu, Japan) at a heating rate of 1°C min^{-1} under air.

Gas adsorption measurements were performed on a BELSORP-max X (MicrotracBEL, Japan). The adsorption/desorption isotherms for N_2 were corrected at 77 K. Before all measurements, the samples were dried under reduced pressure and at 80°C for 3 h.

Proton conductivity testing was performed using pelletized samples pressed in a cylindrical die (surface area: 0.385 cm^2) at 30 MPa for 10 s. The resistance was estimated from the equivalent circuit fitting of Nyquist plots. Proton conductivity was calculated using the equation $\sigma = (1/R)(t/a)$, where σ is the proton conductivity, R is the resistance inside the MOF, and t and a are the thickness and area of the pellet, respectively. Alternating current (AC) impedance measurements were performed using the ALS 760E

dual electrochemical analyzer (BAS Ltd.) in the frequency range $10^0 – 10^6$ Hz with 0.005 V (amplitude voltage). The relative humidity (RH) and temperature were controlled using an IW223 incubator (Yamato Scientific, Japan). The resistance value was determined from the equivalent circuit fits of the first semicircle using the pyZwx software[1]. Direct current (DC) conductivity was determined using a current–potential plot based on the current values obtained upon applying different voltages to the pelletized sample and maintaining them for 60 s.

2.1 Preparation of UiO-66-$(OH)_2$

We prepared UiO-66-$(OH)_2$ with reference to a previous work[2]. $ZrOCl_2 \cdot 8H_2O$ (322 mg, 1.00 mmol, 100 mM) was dissolved in a solution of acetic acid (10 mL) and water (10 mL), and the mixture was sonicated at 20°C for 5 min. Then, 2,5-dihydroxyterephthalic acid (198 mg, 1.00 mmol, 100 mM) was added to the solution and stirred at 20°C for 15 min. Finally, the mixture was placed in a microwave oven and irradiated at 95°C for 15 min. The product was centrifuged at 1986 × g for 20 min, then washed with water, *N*,*N*-dimethylformamide, and methanol, and, lastly, dried at 120°C for 5 h, affording a yellow solid UiO-66-$(OH)_2$ (165 mg).

2.2 Preparation of UiO-66-$(OH)_2$-R

The UiO-66-$(OH)_2$/graphene mesosponge (GMS)/polyvinylidene difluoride (PVdF) composite (20 mg equivalent of UiO-66-$(OH)_2$) was added to a 1 M NH_4HCO_3 aqueous solution (8 mL), and the mixture was sonicated for 30 min to dissolve the UiO-66-$(OH)_2$. The mixture was then filtered, and the same volume of 1 M acetic acid aqueous solution (8 mL) as that of the 1 M NH_4HCO_3 aqueous solution was added to the resulting mixture; the mixture was then concentrated in vacuo. The resulting powder was dispersed in an acetic acid (2 mL) and water (2 mL), and the mixture was sonicated for 15 min. Finally, the mixture was placed in a microwave oven and irradiated at 95°C for 15 min. The product was then centrifuged at 1986 × g for 20 min, washed with water, *N*,*N*-

dimethylformamide, and methanol, and dried at 120°C for 5 h, resulting in a yellow solid UiO-66-$(OH)_2$-R (1.9 mg, 10%).

2.3 Thermogravimetric analysis

As shown in Supplementary Fig. 7, UiO-66-$(OH)_2$ exhibited three-step weight loss: the first step was caused by the loss of absorbed water from the crystal structure, the second step by the dehydration of the Zr clusters, and the third by the organic linker (2,5-dihydroxyterephthalic acid)[3-5]. The combustion reaction (280 – 450°C) of defect-free UiO-66-$(OH)_2$ is represented by the following equation[6]:

$$Zr_6O_6(C_8H_4O_6)_6 + 39O_2 \rightarrow 6ZrO_2 + 48CO_2 + 12H_2O$$

Theoretically, the normalized weight percentage at 280°C was 246.2%, which led to a 24.37% weight loss per organic linker. Experimentally, the normalized weight percentage at 280°C was 244.0%. Therefore, the number of organic linkers containing UiO-66-$(OH)_2$ was calculated as 5.91, *i.e.*, 0.09 organic linker defect per Zr cluster. Since organic linker defect sites were occupied by water and hydroxide anions, the molecular weight of UiO-66-$(OH)_2$ was 1843.9 g mol^{-1} ($Zr_6O_4(OH)_4(C_8H_4O_6)_{5.91}(H_2O)_{0.18}(OH)_{0.09}$)[7].

2.4 Electrode Preparation

UiO-66-$(OH)_2$/Super P/PVdF composite electrodes were prepared by drop-casting a slurry of UiO-66-$(OH)_2$, Super P, and PVdF (4:5:1 w/w/w) and *N*-methyl-2-pyrrolidone

onto glassy carbon substrates. The mass loading of UiO-66-$(OH)_2$ was adjusted to approximately 0.1 – 1.0 mg.

UiO-66-$(OH)_2$/SWNT/PVdF composite electrodes were prepared by coating a slurry of UiO-66-$(OH)_2$, SWNT, and PVdF (4:5:1 w/w/w) and *N*-methyl-2-pyrrolidone onto glassy carbon substrates. The UiO-66-$(OH)_2$/SWNT composite was coated on a glassy carbon plate with a thickness of approximately 10 μm. The mass loading of UiO-66-$(OH)_2$ was adjusted to approximately 0.1 – 1.0 mg.

2.5 Electrochemical Measurements

A tailor-made glass cell (20 cm^2 electrolyte)[8-10] was employed as the electrochemical cell.

2.6 Electrochemical Performance Value Calculation

Coulombic efficiency was calculated according to the following formula:

$$Coulombic\ efficiency = \frac{\sum Q_{discharge}}{\sum Q_{charge}} \times 100$$

$Q_{discharge}$: Charge that flowed when discharging, Q_{charge}: Charge that flowed when charging.

Theoretical capacity was calculated according to the following formula:

$$Theoretical\ capacity = \frac{F\ (As\ mol^{-1}) \times n}{3600\ (s\ h^{-1}) \times M_{\mathrm{MOF}}\ (g\ mol^{-1})} = 171.9\ (mAh\ g^{-1})$$

F: Faraday constant, n: number of redox reacting electrons per unit of UiO-66-$(OH)_2$, M_{MOF}: molecular weight of UiO-66-$(OH)_2$.

2.7 Theoretical calculation

The calculation was performed with the Gaussian 16 package. A representative cluster model of UiO-66-$(OH)_2$ was constructed by truncating the periodic structure around a $Zr_6O_4(OH)_4$ node and capping the terminal carboxylate groups with hydrogen atoms. Geometry optimization was performed only for hydrogen atoms using DFT at the B3LYP level with a mixed basis set (def2-TZVP for Zr and 6-311G(d,p) for C, H, and O) in water using the SMD solvation model. Molecular electrostatic potential (MESP) analysis was then conducted via a single-point calculation using the LANL2DZ basis set and the CHELPG method to obtain atomic charges. MESP plots were displayed by GaussView 6.1.

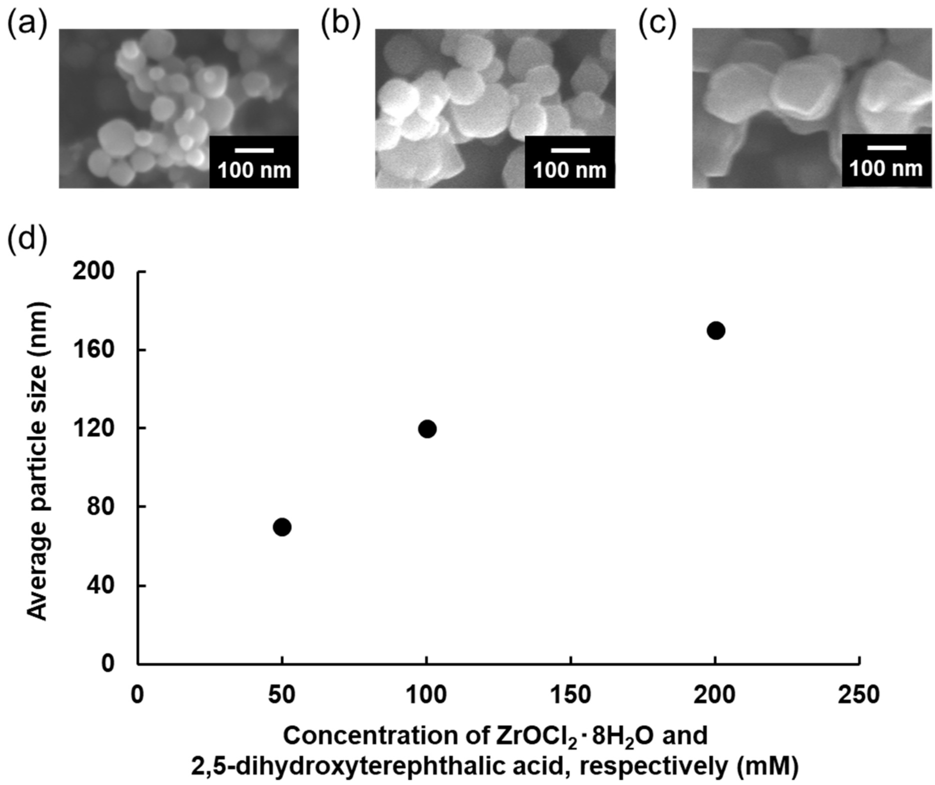

Supplementary Figure 1. **Characterization of UiO-66-$(OH)_2$.** SEM images of UiO-66-$(OH)_2$ prepared at different precursors' concentrations. In each case, concentrations of $ZrOCl_2{\cdot}8H_2O$ and 2,5-dihydroxyterephthalic acid were used: (a) 50 mM, (b) 100 mM, and (c) 200 mM, respectively. The other reaction conditions were identical to those described in the Experimental Section 2.1. (d) The effect of concentrations of $ZrOCl_2{\cdot}8H_2O$ and 2,5-dihydroxyterephthalic acid on the average particle size. Source data are provided as a Source Data file.

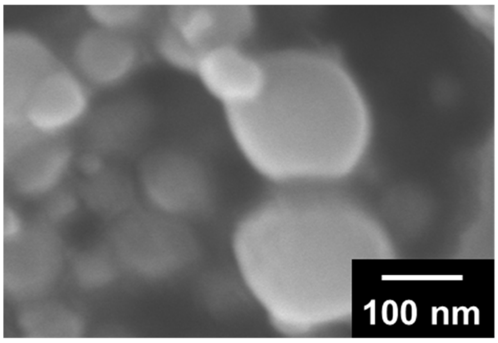

Supplementary Figure 2. **Characterization of UiO-66-$(OH)_2$.** SEM image of UiO-66-$(OH)_2$ with large particle size prepared by long reaction time (scale bar: 100 nm).

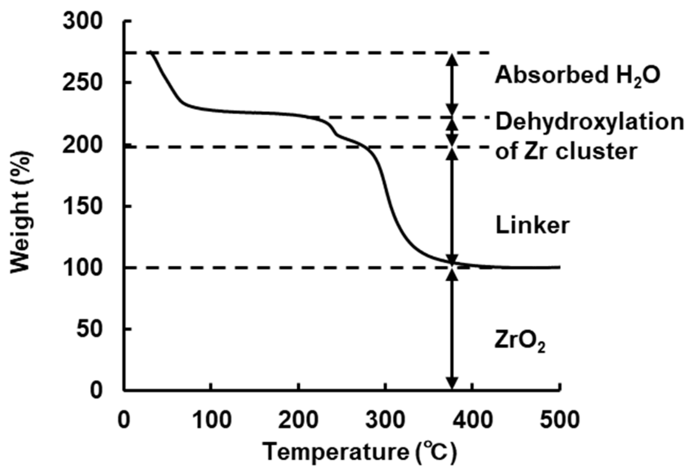

Supplementary Figure 3. Thermogravimetric analysis of UiO-66-(OH)$_2$ with large particle size under air. Theoretically, the normalized weight percentage at 280°C was 246.2%, which led to a 24.37% weight loss per organic linker. Experimentally, the normalized weight percentage at 280°C was 197.7%. Therefore, the number of organic linkers containing UiO-66-(OH)$_2$ was calculated to be 4.01, *i.e.*, 1.99 organic linker defects per Zr cluster. Since organic linker defect sites were occupied by water and hydroxide anions, the molecular weight of UiO-66-(OH)$_2$ was 1571.3 g mol^{-1} ($Zr_6O_4(OH)_4(C_8H_4O_6)_{4.01}(H_2O)_{3.98}(OH)_{1.99}$)[7]. Its theoretical capacity was calculated as 136.8 mAh g^{-1} according to the formula given in the Experimental Section 2.6. According to a previous report, short reaction time decreased the particle size and the number of missing organic linkers[11]. This was likely because a short reaction time suppressed the thermodynamically favorable formation of defects by limiting the competition with acetic acid, resulting in a less defective structure. Source data are provided as a Source Data file.

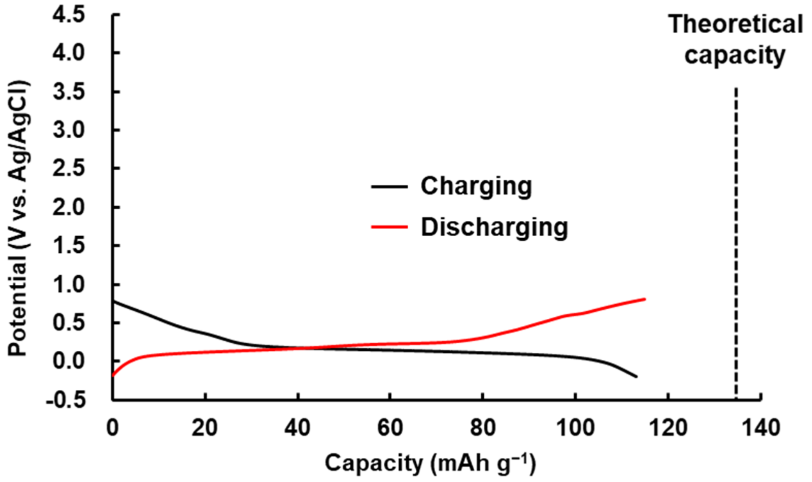

Supplementary Figure 4. Effect of particle size on the electrochemical property of UiO-66-$(OH)_2$ composite electrode. Charging (black) and discharging (red) curves of the UiO-66-$(OH)_2$ (large)/carbon/PVdF composite electrode in a 0.05 M H_2SO_4 aqueous solution of a half-cell at 5 C. Theoretical capacity of UiO-66-$(OH)_2$ (large) was 136.8 mAh g^{-1}. Source data are provided as a Source Data file.

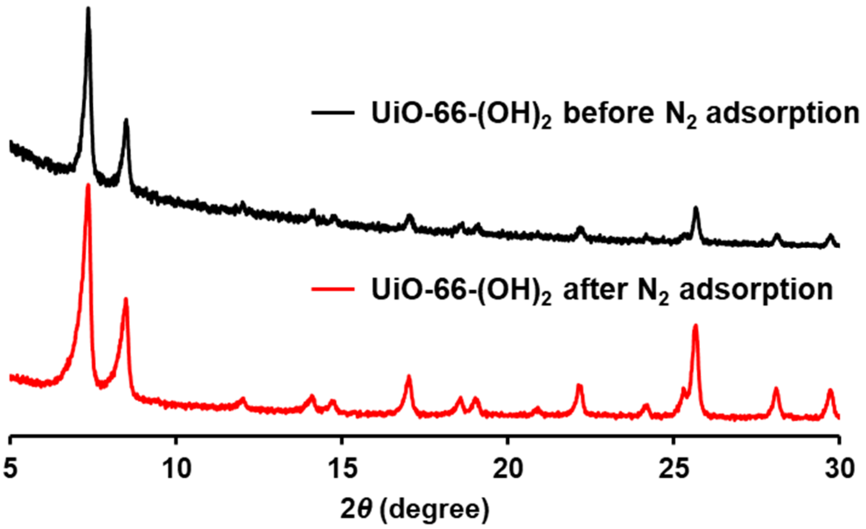

Supplementary Figure 5. Confirmation that the UiO-66-$(OH)_2$ maintains its structure. PXRD patterns of UiO-66-$(OH)_2$ before (black) and after (red) N_2 adsorption measurement. Source data are provided as a Source Data file.

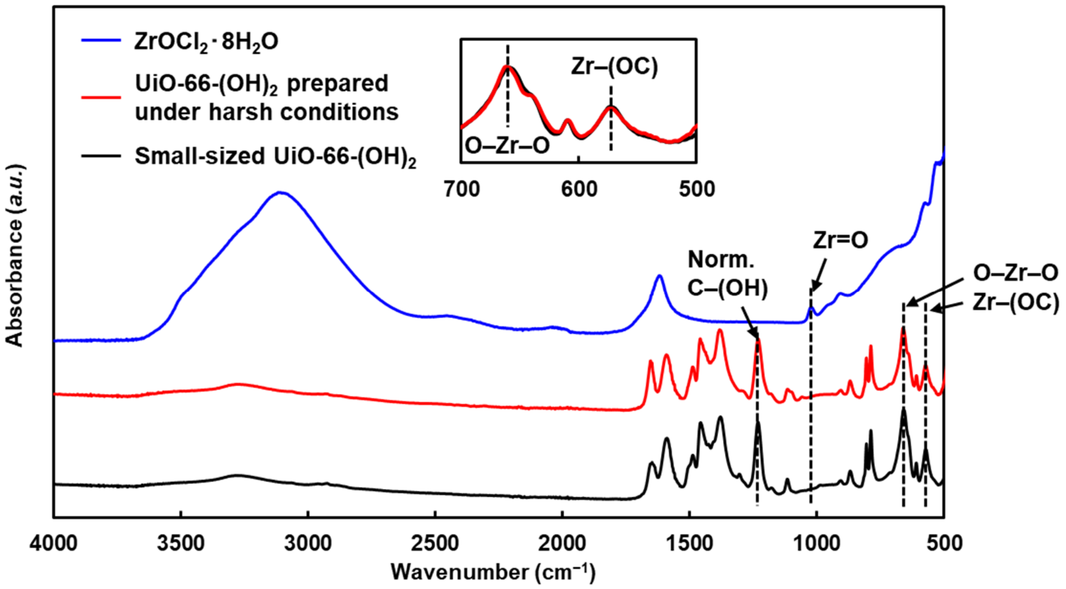

Supplementary Figure 6. **Characterization of UiO-66-$(OH)_2$.** FT-IR spectra of UiO-66-$(OH)_2$ prepared with 95°C, reaction time of 15 min, and lower precursors' concentrations of 50 mM (black, Supplementary Table 1 Entry 2), UiO-66-$(OH)_2$ prepared with higher temperature (110°C), longer reaction time in the microwave (60 min), and higher precursors' concentrations (200 mM) (red), and $ZrOCl_2{\cdot}8H_2O$ (blue). By comparing blue and black lines, $ZrOCl_2$ was almost completely reacted after the preparation reaction of UiO-66-$(OH)_2$, because the peak at 1023 cm^{-1}, derived from Zr=O in $ZrOCl_2{\cdot}8H_2O$[12], disappeared in UiO-66-$(OH)_2$. Inset: The spectra (black and red) were baseline-corrected by specifying three points at 520, 940, and 1280 cm^{-1} and subtracting the linear baseline defined by these points[13,14] because no peaks were observed in these points. After that, the spectra (black and red) were normalized by the peaks at 1235 cm^{-1}, attributed to the C–(OH) of the organic linkers[15-17], which remained unchanged with respect to O–Zr–O and Zr–(OC). Source data are provided as a Source Data file.

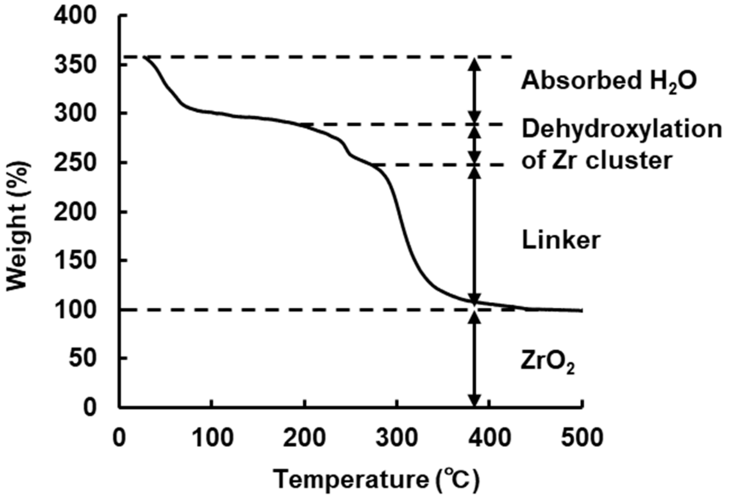

Supplementary Figure 7. **Thermogravimetric analysis of UiO-66-$(OH)_2$ under air.**

Source data are provided as a Source Data file.

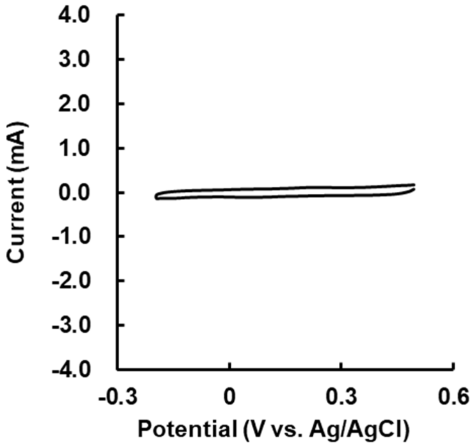

Supplementary Figure 8. Electrochemical performance of the UiO-66-$(OH)_2$/carbon/PVdF composite electrode. Cyclic voltammogram of the UiO-66-$(OH)_2$/carbon/PVdF composite electrode in a 0.05 M H_2SO_4 aqueous solution under an Ar atmosphere at a scan rate of 10 mV s^{-1} before sweeping to positive potential. Source data are provided as a Source Data file.

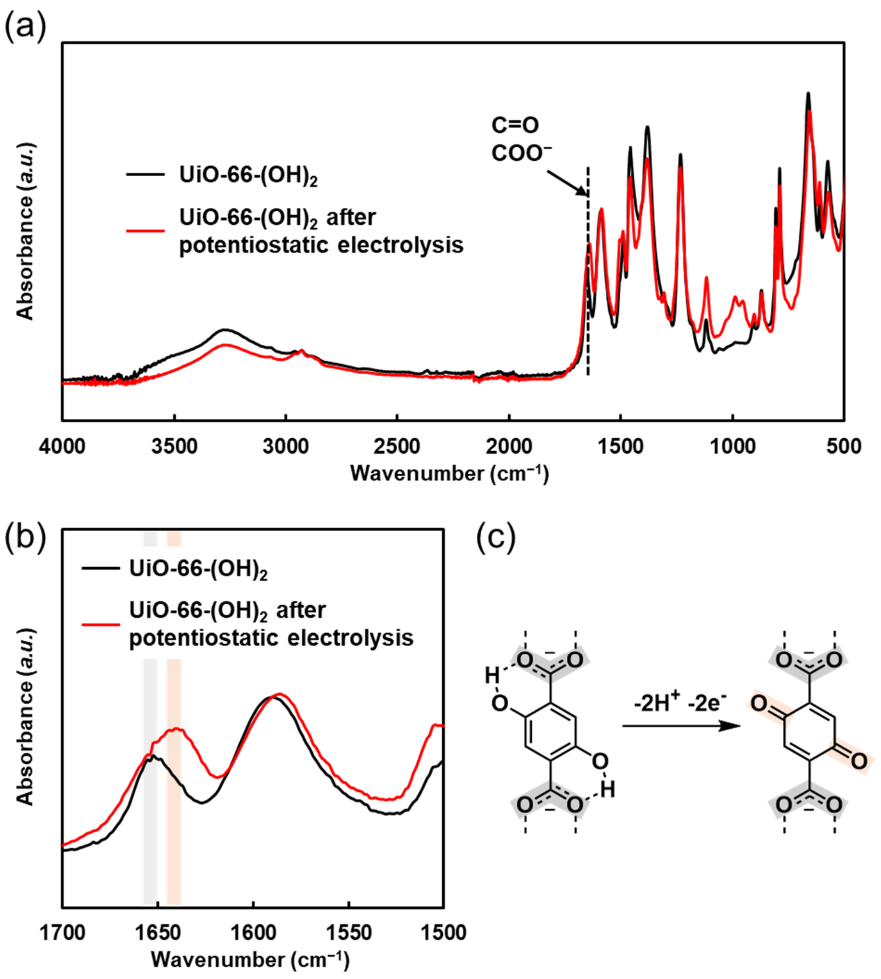

Supplementary Figure 9. **Identification of conformationally changed sites in UiO-66-(OH)$_2$.** (a, b) *Ex situ* FT-IR spectra of UiO-66-(OH)$_2$ (black) and UiO-66-(OH)$_2$ after applying a potential of +0.90 V vs. Ag/AgCl for 2 h in a 0.05 M H_2SO_4 aqueous solution. This spectrum was obtained by converting the transmittance data using the following equation. "$A = \log\frac{1}{T}$ (A: Absorbance, T: Transmittance)" Potentiostatic electrolysis was performed using a UiO-66-(OH)$_2$ electrode, which was fabricated by drop-casting a slurry of UiO-66-(OH)$_2$ and *N*-methyl-2-pyrrolidone onto a glassy carbon electrode and drying at 120°C for 2 h. After performing potentiostatic electrolysis, the electrode was immersed in water to remove the electrolyte for measuring the *ex situ* FT-IR spectrum. The spectra

were normalized by the peaks at 1590 cm^{-1}, attributed to the COO^{-} of the organic linkers[16], which remained unchanged before and after the reaction. (c) Molecular structural changes estimated from the difference of *ex situ* FT-IR spectra. Source data are provided as a Source Data file.

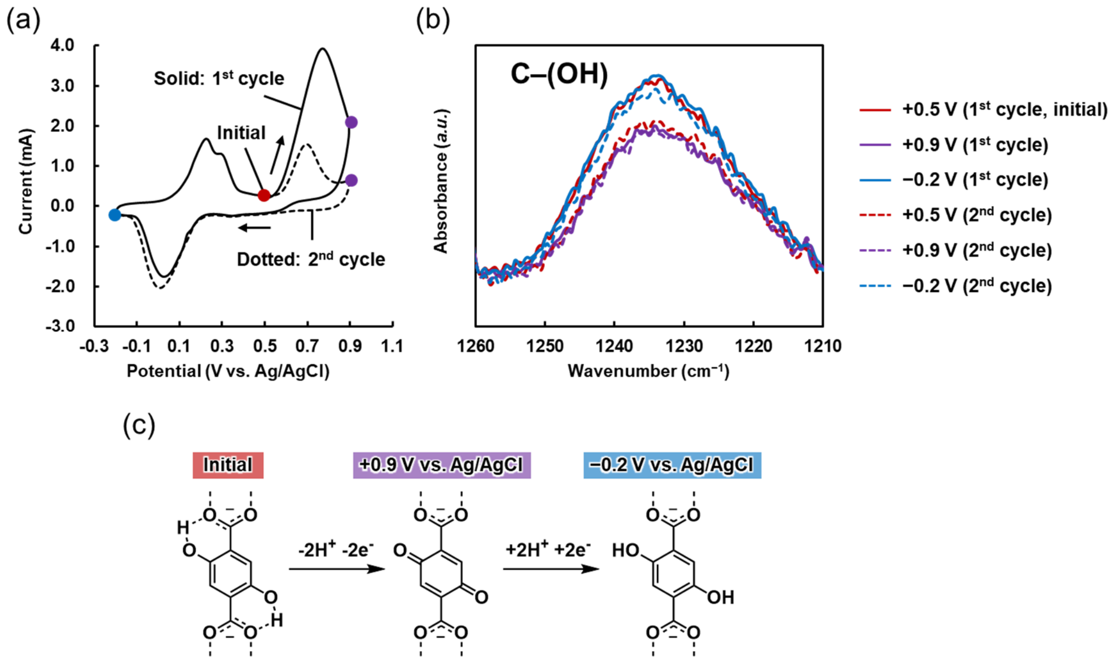

Supplementary Figure 10. **Identification of conformationally changed sites in UiO-66-(OH)$_2$ based on *in situ* measurement.** (a) Different charge/discharge states selected from the cyclic voltammogram and (b) corresponding *in situ* FT-IR spectra. The measurement was performed using an electrode composed of UiO-66-(OH)$_2$ and SWNT (5:1 w/w), in which the carbon ratio is smaller than that described in the Methods. Therefore, a part of the C–(OH) groups of *p*-hydroquinone contributed to the redox reaction. Since a strong peak of H_2O was observed at around 1600 cm^{-1}, we focused on the peak at around 1235 cm^{-1}, corresponding to the C–(OH) group of *p*-hydroquinone[15-17]. *In situ* FT-IR spectra were baseline-corrected by specifying two points at 1210 and 1260 cm^{-1} and subtracting the linear baseline defined by these points[13,14]. (c) The estimated structural changes during charge/discharge measurements. Once the electrode was oxidized, the redox peak in the range of −0.1 – +0.4 V vs. Ag/AgCl became predominant compared to the irreversible oxidation peak at +0.7 V vs. Ag/AgCl (Fig. 2a),

presumably because it took time for the hydrogen bonds to form[18]. Source data are provided as a Source Data file.

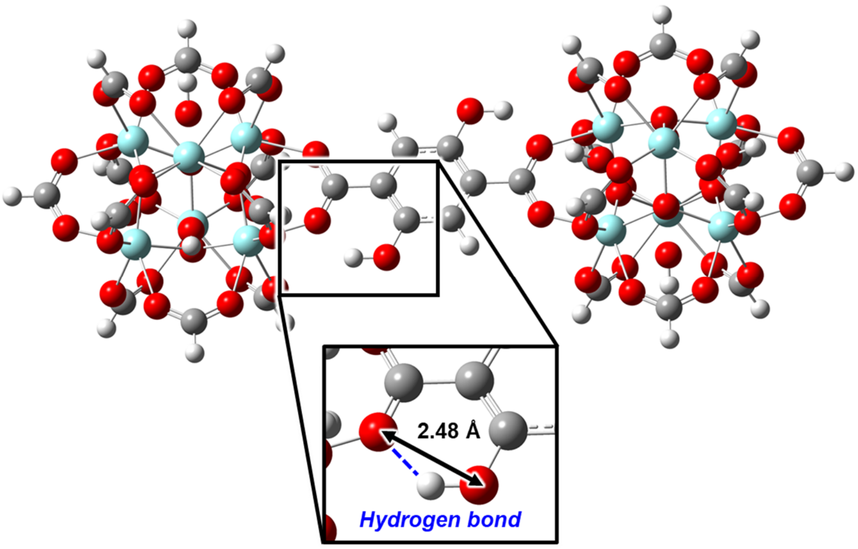

Supplementary Figure 11. **The optimized structure of the cluster of UiO-66-$(OH)_2$.**

The O···O distance was calculated to be 2.48 Å, which indicated the formation of hydrogen bonds between hydroxyl and carboxy groups[19].

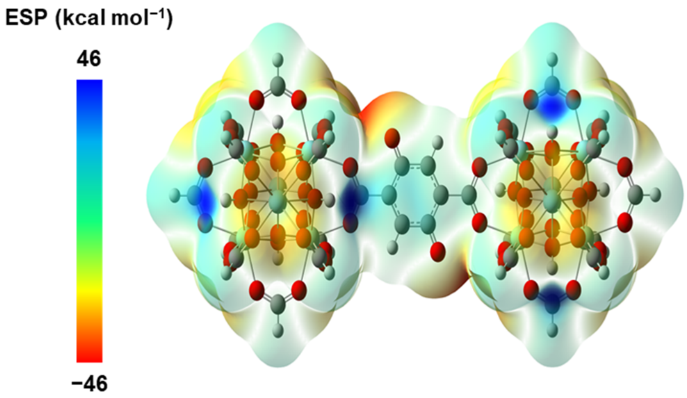

Supplementary Figure 12. **Identification of redox sites in the oxidation state of UiO-66-$(OH)_2$ based on MESP analysis.** On the van der Waals surface of the atoms, the red and blue sites are the electron-rich and electron-deficient regions, respectively. Since it was difficult to take into account whether the hydroxyl groups formed hydrogen bonds with carboxy groups in UiO-66-$(OH)_2$, the calculation was performed for the cluster of the oxidation state of UiO-66-$(OH)_2$ with *p*-benzoquinone as an organic linker.

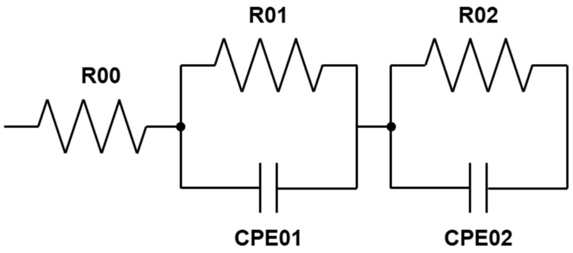

Supplementary Figure 13. **Equivalent circuit for fitting analysis to calculate proton conductivity.** The equivalent circuit considered the resistance of cables and bulk and grain boundary resistances in the impedance measurement. Constant-phase element (CPE) was calculated by following the formula $CPE = \{T(i2\pi f)^{a}\}^{-1}$ $(0 \le \alpha \le 1, i^2 = -1)$, where *T* and *f* were a CPE constant and frequency, respectively.

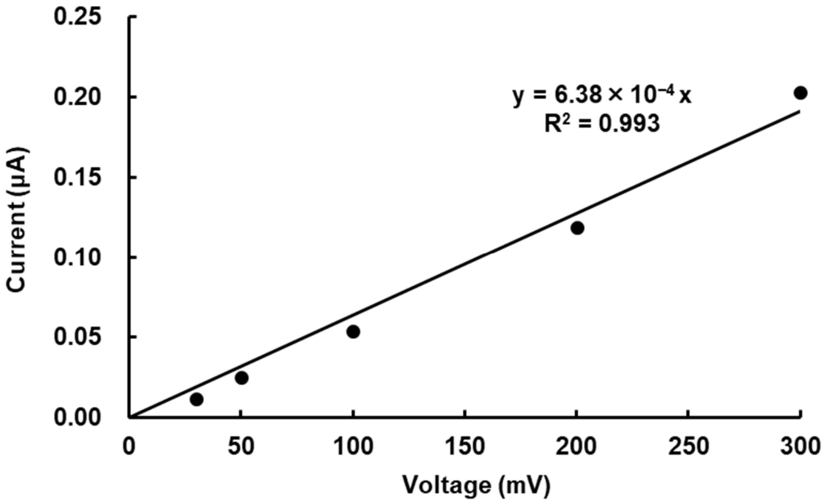

Supplementary Figure 14. DC electrical conductivity measurement of UiO-66-$(OH)_2$.

Current vs. potential of UiO-66-$(OH)_2$ under 95% RH at 30°C. Source data are provided as a Source Data file.

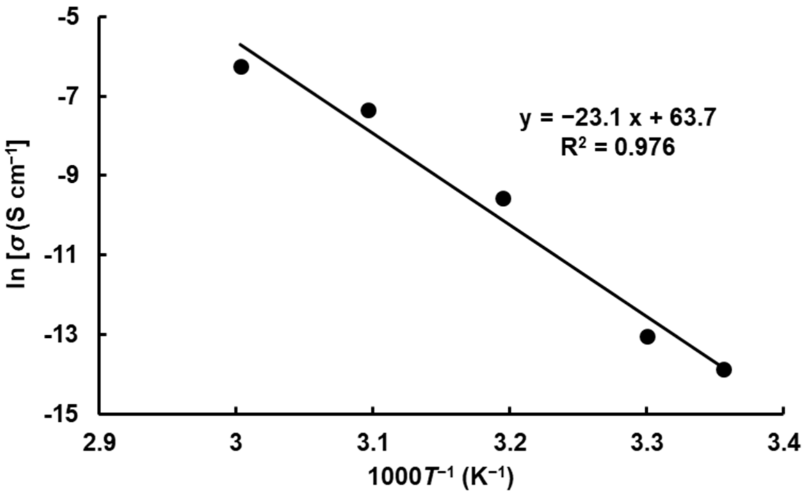

Supplementary Figure 15. Arrhenius plots of proton conductivity of UiO-66-(OH)$_2$.

Arrhenius plots of proton conductivity of UiO-66-(OH)$_2$ under 95% RH at 25, 30, 40, 50, and 60°C. Since UiO-66-(OH)$_2$ had no residual metal ions, under humidified conditions, the possible ionic species are limited to protons (H^+) and hydroxide ions (OH^-). The mobility of hydroxide ions (OH^-) in nanopores via the vehicle mechanism has been reported to be approximately two orders of magnitude lower than that of protons (H^+), due to partial dehydration of hydroxide ions (OH^-) that disrupts their stable hydration shell and requires a large activation energy[20,21]. Therefore, the contribution of hydroxide ions (OH^-) to the ionic conduction of UiO-66-(OH)$_2$ was considered to be much smaller than that of protons (H^+), and the measured ionic conductivity of 2.18×10^{-6} S cm^{-1} was primarily due to proton conduction, not to other types of ions[22]. Source data are provided as a Source Data file.

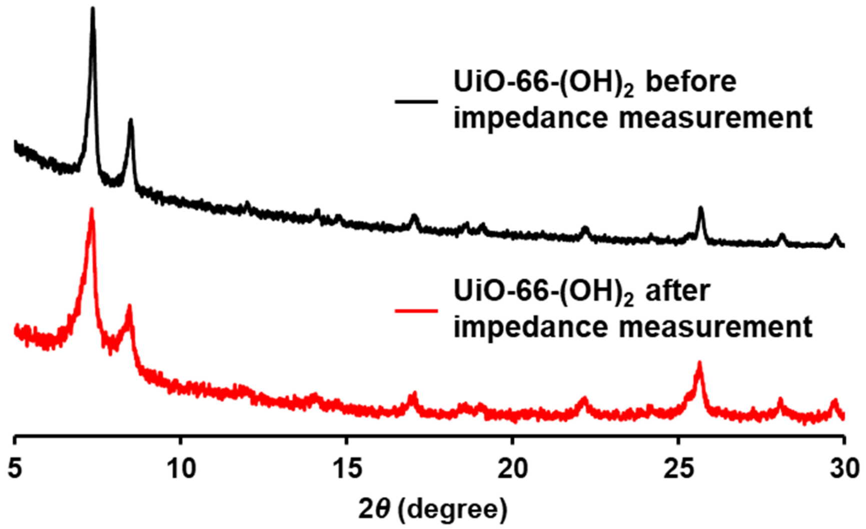

Supplementary Figure 16. Confirmation that the UiO-66-$(OH)_2$ maintains its structure. PXRD patterns of UiO-66-$(OH)_2$ before (black) and after (red) impedance measurement. Source data are provided as a Source Data file.

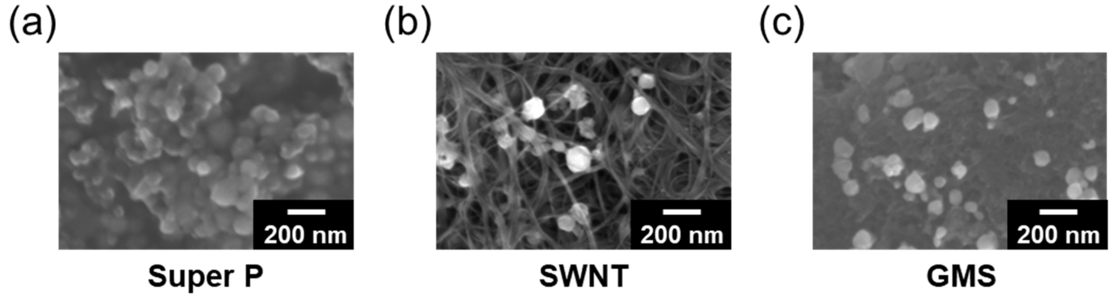

Supplementary Figure 17. Dispersion of UiO-66-$(OH)_2$ in conductive additives. SEM images of (a) UiO-66-$(OH)_2$/Super P/PVdF composite electrode, (b) UiO-66-$(OH)_2$/SWNT/PVdF composite electrode, and (c) UiO-66-$(OH)_2$/GMS/PVdF composite electrode (scale bar: 200 nm).

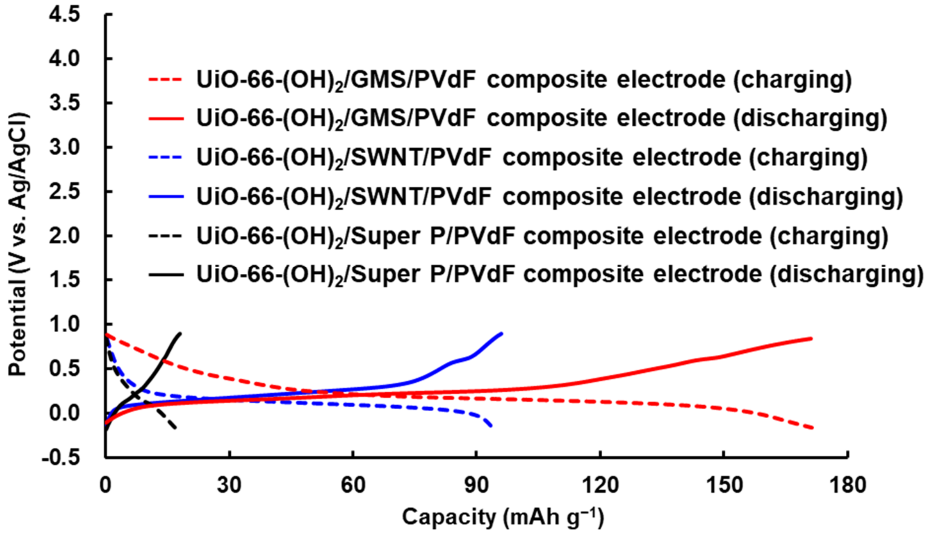

Supplementary Figure 18. Electrochemical performances of UiO-66-(OH)$_2$/carbon/PVdF composite electrodes with different conductive additives. Comparison of the charging (dotted line) and discharging (solid line) curves of UiO-66-(OH)$_2$/GMS/PVdF composite electrode (red), UiO-66-(OH)$_2$/SWNT/PVdF composite electrode (blue), and UiO-66-(OH)$_2$/Super P/PVdF composite electrode (black) in a 0.05 M H_2SO_4 aqueous solution in a half-cell at 5 C. Source data are provided as a Source Data file.

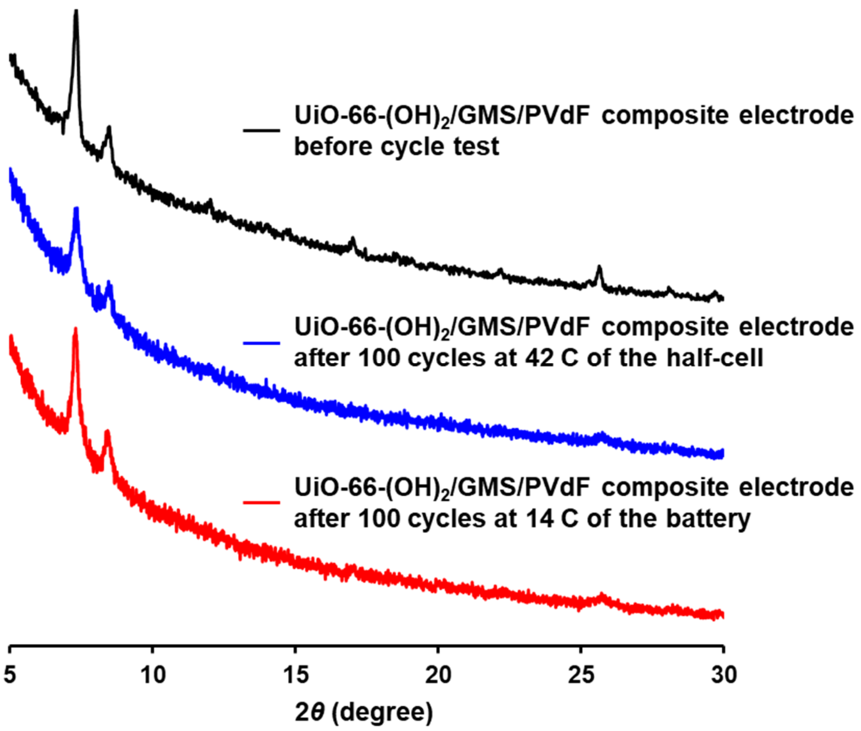

Supplementary Figure 19. Confirmation that the UiO-66-$(OH)_2$ maintains its structure. PXRD patterns of UiO-66-$(OH)_2$/GMS/PVdF composite electrode before cycle test (black), and after 100 cycles of the half-cell (blue) and the battery (red). Source data are provided as a Source Data file.

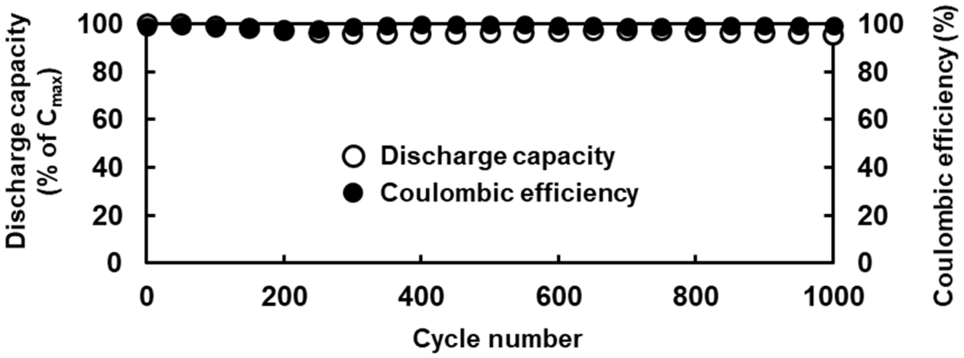

Supplementary Figure 20. The long-term cycle test of the electrode at 45 C. Source data are provided as a Source Data file.

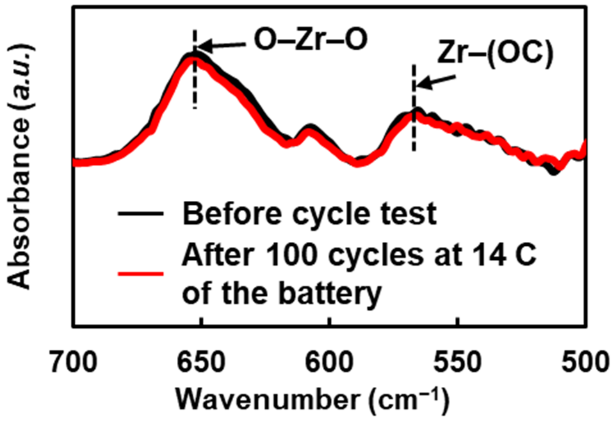

Supplementary Figure 21. Identification of compositional changes in UiO-66-$(OH)_2$. *Ex situ* FT-IR spectra of UiO-66-$(OH)_2$/GMS/PVdF composite electrode before the battery cycle test (black) and after 100 cycles (red). The measurement was performed using an electrode composed of UiO-66-$(OH)_2$, GMS, and PVdF (90:5:10 w/w/w), in which the amount of GMS was smaller than that used in Figs. 2 and 3, because GMS readily absorbed infrared light. *Ex situ* FT-IR spectra were baseline-corrected by specifying three points at 520, 590, and 700 cm^{-1} and subtracting the linear baseline defined by these points[13,14]. Source data are provided as a Source Data file.

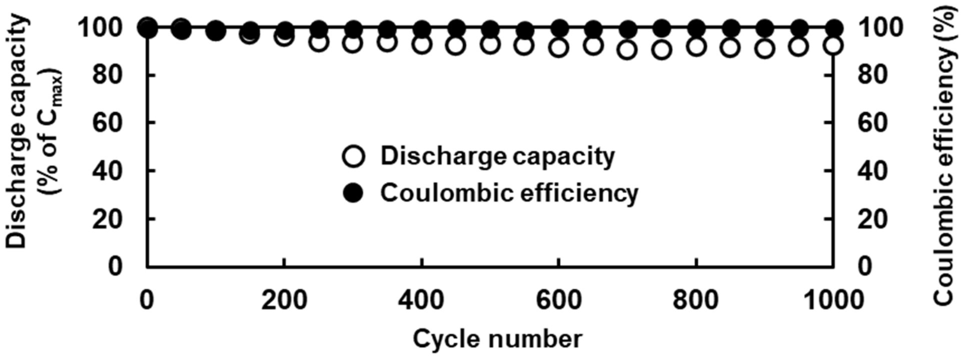

Supplementary Figure 22. The long-term battery cycle test at 10 C. Source data are provided as a Source Data file.

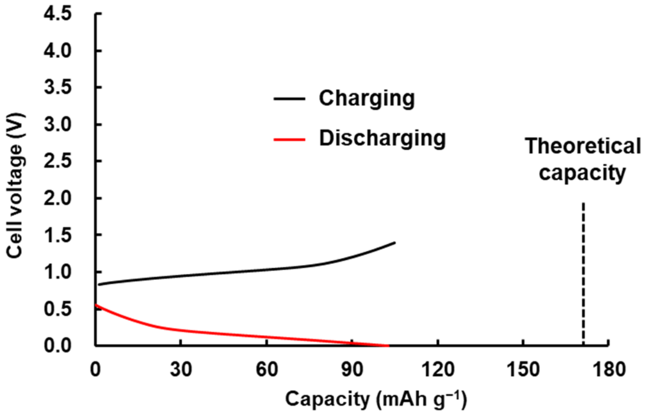

Supplementary Figure 23. High-rate capability of the battery. Charging (black)/discharging (red) curves of the battery at 60 C. The dotted line represents the theoretical capacity based on the molecular weight of UiO-66-$(OH)_2$ (171.9 mAh g^{-1}). Source data are provided as a Source Data file.

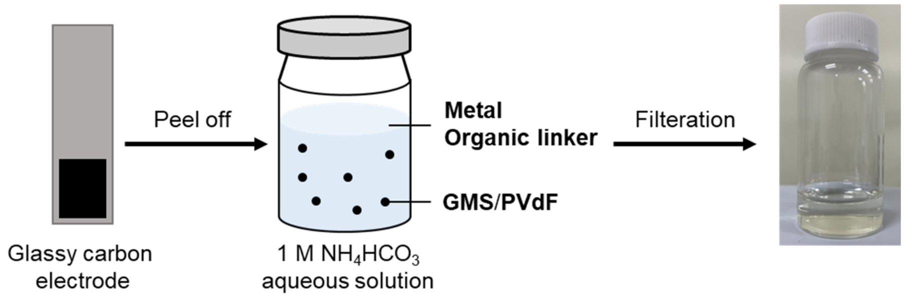

Supplementary Figure 24. **Schematic diagram of UiO-66-$(OH)_2$ decomposition.** The UiO-66-$(OH)_2$/GMS/PVdF composite electrode was peeled off with a spatula. The electrode remained firmly adhered during actual battery operation, and no detachment was observed during electrochemical testing.

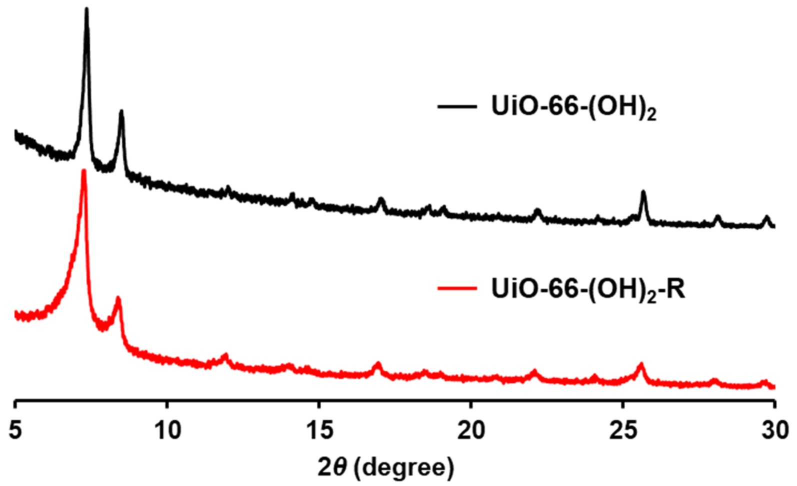

Supplementary Figure 25. Characterization of UiO-66-$(OH)_2$ after recycling. PXRD patterns of UiO-66-$(OH)_2$ (black) and UiO-66-$(OH)_2$-R (red). Source data are provided as a Source Data file.

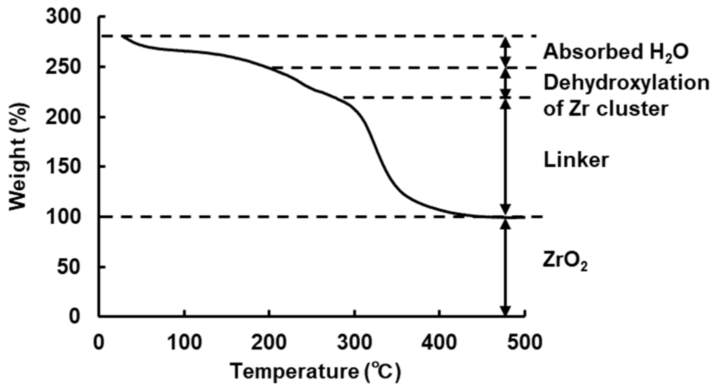

Supplementary Figure 26. **Thermogravimetric analysis of UiO-66-(OH)$_2$-R under air.** Based on the TGA profile, the number of organic linkers containing UiO-66-(OH)$_2$-R was 4.87, *i.e.,* 1.13 organic linker defects per Zr cluster. Since organic linker defect sites were occupied by water and hydroxide anions, the molecular weight of UiO-66-(OH)$_2$-R was 1694.4 g mol^{-1} ($Zr_6O_4(OH)_4(C_8H_4O_6)_{4.87}(H_2O)_{2.26}(OH)_{1.13}$)[7]. The theoretical capacity calculated from the molecular weight of UiO-66-(OH)$_2$-R was 154.1 mAh g^{-1}. The details of the calculation are described in the Experimental Section 2.3 and 2.6. Source data are provided as a Source Data file.

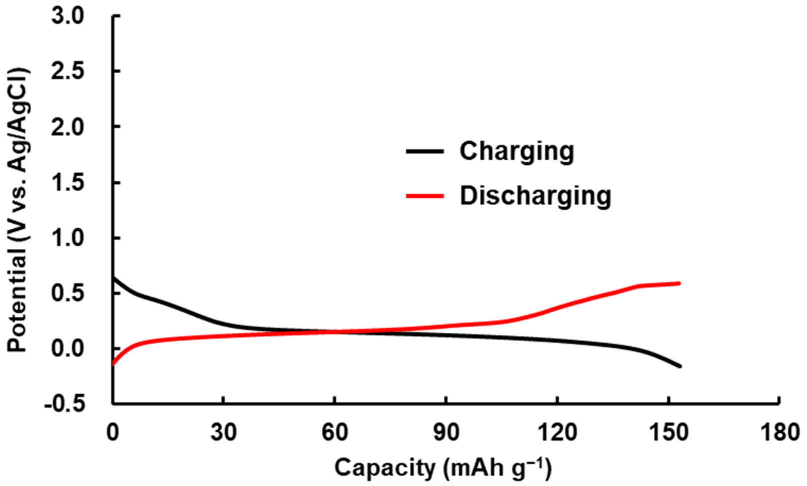

Supplementary Figure 27. Electrochemical performance of the UiO-66-$(OH)_2$-R/GMS/PVdF composite electrode. Charging (black) and discharging (red) curves of the UiO-66-$(OH)_2$-R/GMS/PVdF composite electrode in a half-cell at 5 C. Source data are provided as a Source Data file.1

Supplementary Table 1. **Synthetic conditions of UiO-66-$(OH)_2$.**

Entry	Ref. No.	Reaction time (min)	$ZrOCl_2 \cdot 8H_2O$ (mmol)	2,5-Dihydroxy terephthalic acid (mmol)	Water (mL)	Acetic acid (mL)	Average particle size[a] (nm)
1	[2]	60	4.0	4.0	10	10	Not Reported
2	This work	15	1.0	1.0	10	10	70 ± 20
3	This work	60	1.0	1.0	10	10	120 ± 40

[a] Estimated from SEM images.

Supplementary Table 2. Fitting parameters of proton conductivity measurement under 95% RH at 30°C.

R00 (Err.)	R01 (Err.)	CPE01_T (Err.)	CPE01_a (Err.)	R02 (Err.)	CPE02_T (Err.)	CPE02_a (Err.)
9.69 (8.52×10)	2.74×10^{4} (7.01×10^{2})	6.44×10^{-10} (3.90×10^{-10})	8.29×10^{-1} (2.39×10^{-3})	3.11×10^{10} (1.50×10^{14})	1.20×10^{-6} (6.53×10^{-8})	5.65×10^{-1} (1.24×10^{-2})

Supplementary Table 3. Full-cell performances of aqueous MOF-based rechargeable batteries.

Ref. No.	Anode-active materials	Cathode-active materials	Electrolyte (aqueous solution)	Discharge capacity/ Theoretical capacity (%)	Discharge capacity retention after 100 cycles (%)	Coulombic efficiency (%)
**This work**	**UiO-66-(OH)$_2$**	**O_2**	**0.05 M H_2SO_4**	**99.9**	**99**	**99.9**
[23]	Zn	Ni-Ndi-trz	2 M $ZnSO_4$	72[a]	93	99
[24]	Zn	Mn-MOF-74	2 M $ZnSO_4$	80[b]	79	98
[25]	Zn	$Cu_3(HHTP)_2$	3 M $Zn(CF_3SO_3)_2$	77[c]	75	99
[26]	Zn	Cu-BTA	2.5 M $ZnSO_4$	96	87	97
[27]	Zn	MIL-100(V)	1 M $Zn(CF_3SO_3)_2$	93[d]	99	99

[a] Calculated based on 2 electrons charging in one unit (419.9 g mol^{-1}).

[b] Calculated based on 4 electrons charging in one unit (338.0 g mol^{-1}).

[c] Calculated based on 3 electrons charging in one unit (275.7 g mol^{-1}).

[d] Calculated based on 9 electrons charging in one unit (623.1 g mol^{-1}).

In some cases, the capacities clearly exceed the theoretical capacities calculated from redox reactions, but since the causes have not been discussed, they are excluded from the discussion in this paper[28,29].

Although performance between RAMOFs should be compared with half-cell measurements, no data were exhibited in the papers, and all data were based on full-cell measurements with zinc as the anode. Therefore, in the current work, the performance of RAMOFs was compared with batteries.

Supplementary Table 4. Full-cell performances of aqueous organic–air rechargeable batteries.

Ref. No.	Anode-active materials	Cathode-active materials	Electrolyte (aqueous solution)	Discharge capacity/ Theoretical capacity (%)	Discharge capacity retention after 100 cycles (%)	Coulombic efficiency[a] (%)
**This work**	**UiO-66-$(OH)_2$**	**Pt/C**	**0.05 M H_2SO_4**	**99.9**	**99**	**99.9**
[8]	PDBM 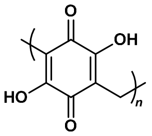	Pt/C	H_2SO_4 (pH 1)	98	96	99
[30]	P14AQ 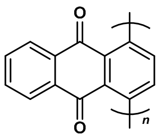	Spinel cobalt manganese oxide supported on CNT	6 M KOH	97	96	95
[31]	PVAQ 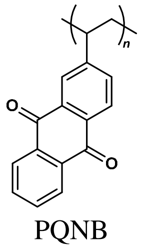	VGCF, MnO_2, and PVdF	30 wt% KOH	93	87	93
[32]	PQNB 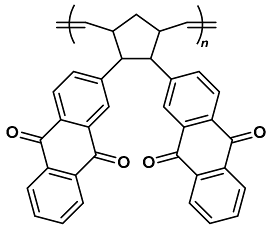	VGCF, MnO_2, and PVdF	10 M NaOH	99	97	99
[10]	pEP(NQ)E 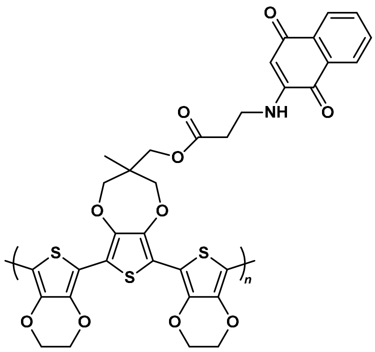	Pt/C	H_2SO_4 (pH 1)	99	98	99

[9]	PNQ 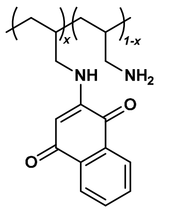	Pt/C	0.5 M H_2SO_4	99	93	99
[33]	PDAQ 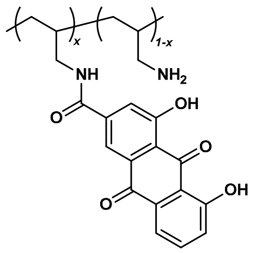	Pt/C	0.5 M H_2SO_4 in air	95	98	99
[34]	AQ-CMP 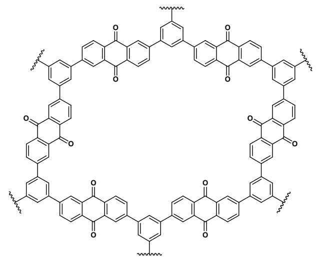	Pt/C	6 M KOH	96	99	95
[35]	0.4EDOT@TpOMe-DAQ 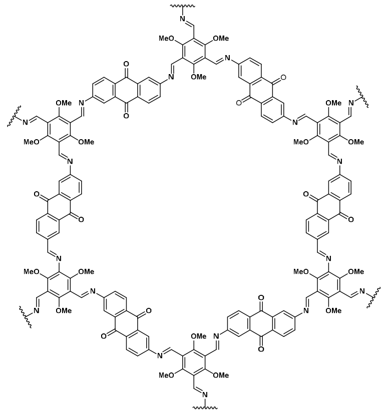	Pt/C	0.5 M H_2SO_4	97	99	97
[36]	BBL 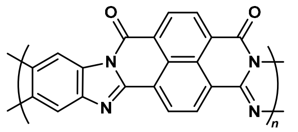	Pt/C	0.5 M H_2SO_4	93	99	95
[37]	CO-POSSs with AQ 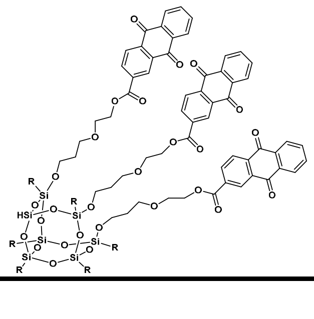	Pt/C	0.1 M NaOH	35	90	91

[38]	NHCC 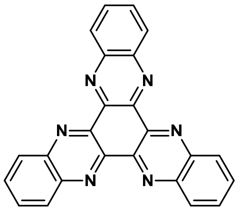	D-CoPPc, AB, PVdF on hydrophobic carbon cloth	4 M KOH + 10 M KCF_3SO_3	60	93	76

[a] If there is a variation in Coulombic efficiency or capacity retention, the minimum value is displayed. CNT: Carbon nanotube, VGCF: Vapor-grown carbon fiber, AB: Acetylene black.

Supplementary Table 5. Cartesian coordinates of optimized geometries of Supplementary Figure 11.

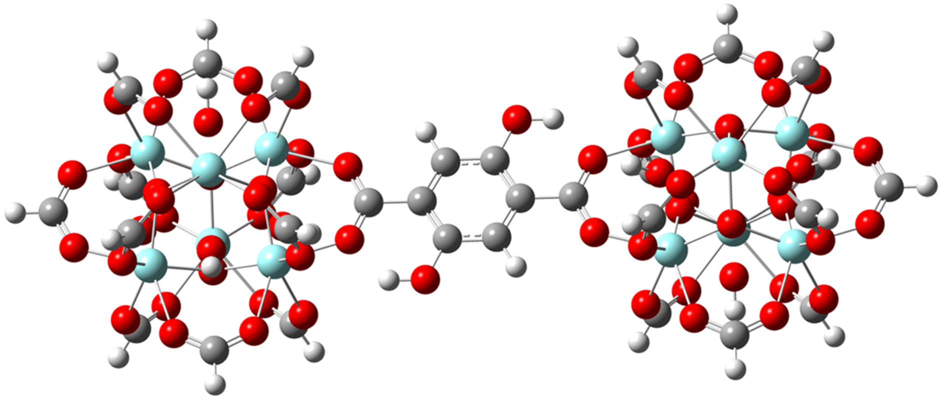

Atom	X	Y	Z
O	-2.51262	-1.947717	4.828592
C	-2.23012	-3.146026	5.111733
O	-1.3837	-3.546054	5.959951
Zr	-0.00781	-2.4761	7.338704
Zr	-1.75675	0.000058	5.585993
O	-2.5125	1.947883	4.828592
C	-2.22991	3.146174	5.111733
O	-1.38346	3.546146	5.959951
Zr	-0.00765	2.4761	7.338704
O	-2.50245	-1.947717	-4.83388
C	-2.21935	-3.146026	-5.11642
O	-1.37114	-3.546054	-5.96285
Zr	0.007648	-2.4761	-7.3387
Zr	-1.74498	0.000058	-5.58968
O	-2.50232	1.947883	-4.83388
C	-2.21914	3.146174	-5.11642
O	-1.37091	3.546146	-5.96285
Zr	0.007813	2.4761	-7.3387
Zr	1.74498	-0.000058	5.589682
O	2.502445	1.947717	4.833875
C	2.219345	3.146026	5.11642
O	1.37114	3.546054	5.962853
Zr	1.741291	-0.000058	9.091414
O	2.502316	-1.947883	4.833875
O	1.12653	-0.000037	3.455122
O	3.878232	-0.000129	6.212628

Zr	-1.76044	0.000058	9.087725
O	-2.51791	-1.947717	9.843532
C	-2.23481	-3.146026	9.560988
O	-1.3866	-3.546054	8.714555
O	-2.51778	1.947883	9.843532
O	-3.89369	0.000129	8.46478
O	-1.14199	0.000038	11.22229
C	-2.2346	3.146174	9.560988
O	-1.38637	3.546146	8.714555
C	2.219136	-3.146174	5.11642
O	1.370905	-3.546146	5.962853
O	1.368238	3.546054	8.717456
C	2.214658	3.146026	9.565675
O	2.497163	1.947717	9.848815
O	1.368003	-3.546146	8.717456
C	2.214449	-3.146174	9.565674
O	2.497033	-1.947883	9.848815
O	-3.87823	0.000129	-6.21263
C	-4.44152	0.000147	-7.34339
O	-3.87585	0.000129	-8.47296
Zr	-1.74129	0.000058	-9.09141
O	-3.89131	0.000129	6.204443
C	-4.45699	0.000148	7.334017
O	-1.13381	0.000038	3.452741
C	-0.00304	0	2.889449
C	-0.00146	0	1.385858
C	1.189541	-0.000039	0.695491
C	-1.191	0.000039	0.692983
H	-2.13409	-0.000899	1.222212
O	-1.12653	0.000037	-3.45512
C	0.003044	0	-2.88945
C	0.00146	0	-1.38586
C	1.191003	-0.000039	-0.69298
H	2.134091	0.000899	-1.22221
O	1.133806	-0.000038	-3.45274
C	-1.18954	0.000039	-0.69549
Zr	1.756752	-0.000058	-5.58599
C	-0.01242	0	11.78796
O	1.118345	-0.000037	11.22467

C	4.441524	-0.000147	7.34339
O	3.875851	-0.000129	8.472964
O	2.512624	1.947717	-4.82859
C	2.230119	3.146026	-5.11173
O	1.383699	3.546054	-5.95995
O	2.512495	-1.947883	-4.82859
C	2.22991	-3.146174	-5.11173
O	1.383464	-3.546146	-5.95995
Zr	1.760441	-0.000058	-9.08773
O	-1.368	3.546146	-8.71746
O	1.386601	3.546054	-8.71456
O	-1.36824	-3.546054	-8.71746
C	-2.21466	-3.146026	-9.56568
O	-2.49716	-1.947717	-9.84882
O	1.386366	-3.546146	-8.71456
C	-2.21445	3.146174	-9.56567
O	-2.49703	1.947883	-9.84882
O	3.891312	-0.000129	-6.20444
C	4.456985	-0.000148	-7.33402
O	3.893693	-0.000129	-8.46478
O	-1.11835	0.000037	-11.2247
C	0.012417	0	-11.788
O	1.141991	-0.000038	-11.2223
C	2.234806	3.146026	-9.56099
O	2.517906	1.947717	-9.84353
C	2.234597	-3.146174	-9.56099
O	2.517777	-1.947883	-9.84353
O	1.471854	1.046151	7.340262
O	-0.00932	-1.0462	8.818253
O	-1.48725	1.046249	7.337145
O	-0.00621	-1.0462	5.859154
O	-0.00559	1.4032	5.35428
H	-0.00474	1.9615	4.564725
O	-1.9922	-1.403134	7.336613
H	-2.78177	-1.961408	7.335782
O	1.976646	-1.403266	7.340794
H	2.766183	-1.961592	7.341626
O	-0.00977	1.4032	9.323127
H	-0.01059	1.9615	10.11268

O	1.9922	1.403134	-7.33661
H	2.781774	1.961408	-7.33578
O	0.005594	-1.4032	-5.35428
H	0.004743	-1.9615	-4.56473
O	-1.97665	1.403266	-7.34079
H	-2.76618	1.961592	-7.34163
O	0.009774	-1.4032	-9.32313
H	0.010588	-1.9615	-10.1127
O	0.009324	1.0462	-8.81825
O	-1.47185	-1.046151	-7.34026
O	1.487245	-1.046249	-7.33715
O	0.006207	1.0462	-5.85915
O	2.439671	0.003663	1.346675
O	-2.43967	-0.003663	-1.34668
H	-2.75493	-3.902613	-4.58198
H	-2.75467	3.902796	-4.58198
H	-2.74886	3.902796	-10.1012
H	0.013544	0	-12.858
H	2.770135	-3.902796	-10.0954
H	2.764321	-3.902796	-4.57617
H	5.526985	-0.000183	-7.33289
H	2.770394	3.902613	-10.0954
H	2.76458	3.902613	-4.57617
H	-5.51152	0.000183	-7.34452
H	-2.74912	-3.902613	-10.1012
H	5.511524	-0.000183	7.344518
H	2.754933	3.902613	4.581984
H	2.749119	3.902613	10.10124
H	-0.01354	0	12.85796
H	-2.77014	3.902796	10.09542
H	-2.76432	3.902796	4.57617
H	-2.76458	-3.902613	4.576169
H	-5.52699	0.000183	7.33289
H	-2.77039	-3.902613	10.09542
H	2.754674	-3.902796	4.581983
H	2.74886	-3.902796	10.10124
H	2.426135	-0.000136	2.371836
H	-2.42614	0.000136	-2.37184

Supplementary Table 6. Cartesian coordinates of optimized geometries of Supplementary Figure 12.

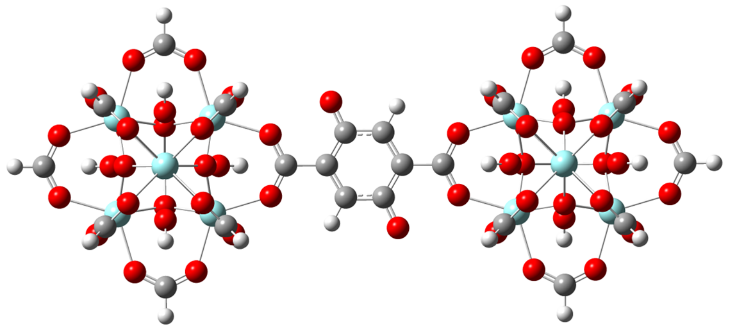

Atom	X	Y	Z
O	-2.51262	-1.947717	4.828592
C	-2.23012	-3.146026	5.111733
O	-1.3837	-3.546054	5.959951
Zr	-0.00781	-2.4761	7.338704
Zr	-1.75675	0.000058	5.585993
O	-2.5125	1.947883	4.828592
C	-2.22991	3.146174	5.111733
O	-1.38346	3.546146	5.959951
Zr	-0.00765	2.4761	7.338704
O	-2.50245	-1.947717	-4.83388
C	-2.21935	-3.146026	-5.11642
O	-1.37114	-3.546054	-5.96285
Zr	0.007648	-2.4761	-7.3387
Zr	-1.74498	0.000058	-5.58968
O	-2.50232	1.947883	-4.83388
C	-2.21914	3.146174	-5.11642
O	-1.37091	3.546146	-5.96285
Zr	0.007813	2.4761	-7.3387
Zr	1.74498	-0.000058	5.589682
O	2.502445	1.947717	4.833875
C	2.219345	3.146026	5.11642
O	1.37114	3.546054	5.962853
Zr	1.741291	-0.000058	9.091414
O	2.502316	-1.947883	4.833875
O	1.12653	-0.000037	3.455122
O	3.878232	-0.000129	6.212628
Zr	-1.76044	0.000058	9.087725

O	-2.51791	-1.947717	9.843532
C	-2.23481	-3.146026	9.560988
O	-1.3866	-3.546054	8.714555
O	-2.51778	1.947883	9.843532
O	-3.89369	0.000129	8.46478
O	-1.14199	0.000038	11.22229
C	-2.2346	3.146174	9.560988
O	-1.38637	3.546146	8.714555
C	2.219136	-3.146174	5.11642
O	1.370905	-3.546146	5.962853
O	1.368238	3.546054	8.717456
C	2.214658	3.146026	9.565675
O	2.497163	1.947717	9.848815
O	1.368003	-3.546146	8.717456
C	2.214449	-3.146174	9.565674
O	2.497033	-1.947883	9.848815
O	-3.87823	0.000129	-6.21263
C	-4.44152	0.000147	-7.34339
O	-3.87585	0.000129	-8.47296
Zr	-1.74129	0.000058	-9.09141
O	-3.89131	0.000129	6.204443
C	-4.45699	0.000148	7.334017
O	-1.13381	0.000038	3.452741
C	-0.00304	0	2.889449
C	-0.00146	0	1.385858
C	1.189541	-0.000039	0.695491
C	-1.191	0.000039	0.692983
H	-2.14123	-0.000048	1.207273
O	-1.12653	0.000037	-3.45512
C	0.003044	0	-2.88945
C	0.00146	0	-1.38586
C	1.191003	-0.000039	-0.69298
H	2.141234	0.000048	-1.20727
O	1.133806	-0.000038	-3.45274
C	-1.18954	0.000039	-0.69549
Zr	1.756752	-0.000058	-5.58599
C	-0.01242	0	11.78796
O	1.118345	-0.000037	11.22467
C	4.441524	-0.000147	7.34339
O	3.875851	-0.000129	8.472964

O	2.512624	1.947717	-4.82859
C	2.230119	3.146026	-5.11173
O	1.383699	3.546054	-5.95995
O	2.512495	-1.947883	-4.82859
C	2.22991	-3.146174	-5.11173
O	1.383464	-3.546146	-5.95995
Zr	1.760441	-0.000058	-9.08773
O	-1.368	3.546146	-8.71746
O	1.386601	3.546054	-8.71456
O	-1.36824	-3.546054	-8.71746
C	-2.21466	-3.146026	-9.56568
O	-2.49716	-1.947717	-9.84882
O	1.386366	-3.546146	-8.71456
C	-2.21445	3.146174	-9.56567
O	-2.49703	1.947883	-9.84882
O	3.891312	-0.000129	-6.20444
C	4.456985	-0.000148	-7.33402
O	3.893693	-0.000129	-8.46478
O	-1.11835	0.000037	-11.2247
C	0.012417	0	-11.788
O	1.141991	-0.000038	-11.2223
C	2.234806	3.146026	-9.56099
O	2.517906	1.947717	-9.84353
C	2.234597	-3.146174	-9.56099
O	2.517777	-1.947883	-9.84353
O	1.471854	1.046151	7.340262
O	-0.00932	-1.0462	8.818253
O	-1.48725	1.046249	7.337145
O	-0.00621	-1.0462	5.859154
O	-0.00559	1.4032	5.35428
H	-0.00474	1.9615	4.564725
O	-1.9922	-1.403134	7.336613
H	-2.78177	-1.961408	7.335782
O	1.976646	-1.403266	7.340794
H	2.766183	-1.961592	7.341626
O	-0.00977	1.4032	9.323127
H	-0.01059	1.9615	10.11268
O	1.9922	1.403134	-7.33661
H	2.781774	1.961408	-7.33578
O	0.005594	-1.4032	-5.35428

H	0.004743	-1.9615	-4.56473
O	-1.97665	1.403266	-7.34079
H	-2.76618	1.961592	-7.34163
O	0.009774	-1.4032	-9.32313
H	0.010588	-1.9615	-10.1127
O	0.009324	1.0462	-8.81825
O	-1.47185	-1.046151	-7.34026
O	1.487245	-1.046249	-7.33715
O	0.006207	1.0462	-5.85915
O	2.318662	0.002128	1.221481
O	-2.31866	-0.002128	-1.22148
H	-2.75493	-3.902613	-4.58198
H	-2.75467	3.902796	-4.58198
H	-2.74886	3.902796	-10.1012
H	0.013544	0	-12.858
H	2.770135	-3.902796	-10.0954
H	2.764321	-3.902796	-4.57617
H	5.526985	-0.000183	-7.33289
H	2.770394	3.902613	-10.0954
H	2.76458	3.902613	-4.57617
H	-5.51152	0.000183	-7.34452
H	-2.74912	-3.902613	-10.1012
H	5.511524	-0.000183	7.344518
H	2.754933	3.902613	4.581984
H	2.749119	3.902613	10.10124
H	-0.01354	0	12.85796
H	-2.77014	3.902796	10.09542
H	-2.76432	3.902796	4.57617
H	-2.76458	-3.902613	4.576169
H	-5.52699	0.000183	7.33289
H	-2.77039	-3.902613	10.09542
H	2.754674	-3.902796	4.581983
H	2.74886	-3.902796	10.10124

References

1. Kobayashi, K. & Suzuki, T. S. Free Analysis and Visualization Programs for Electrochemical Impedance Spectroscopy Coded in Python. *Electrochemistry* **89**, 218-222 (2021).
2. Reinsch, H., Waitschat, S., Chavan, S. M., Lillerud, K. P. & Stock, N. A Facile "Green" Route for Scalable Batch Production and Continuous Synthesis of Zirconium MOFs. *Eur. J. Inorg. Chem.* **2016**, 4490-4498 (2016).
3. Valenzano, L. *et al.* Disclosing the Complex Structure of UiO-66 Metal Organic Framework: A Synergic Combination of Experiment and Theory. *Chem. Mater.* **23**, 1700-1718 (2011).
4. Taylor, J. M., Dekura, S., Ikeda, R. & Kitagawa, H. Defect Control To Enhance Proton Conductivity in a Metal–Organic Framework. *Chem. Mater.* **27**, 2286-2289 (2015).
5. Haidar, O. *et al.* Defect-Rich Metal–Organic Framework Nanocrystals for Removal of Micropollutants from Water. *ACS Appl. Nano Mater.* **7**, 10003-10015 (2024).
6. Shearer, G. C. *et al.* Defect Engineering: Tuning the Porosity and Composition of the Metal–Organic Framework UiO-66 via Modulated Synthesis. *Chem. Mater.* **28**, 3749-3761 (2016).
7. Trickett, C. A. *et al.* Definitive Molecular Level Characterization of Defects in UiO-66 Crystals. *Angew. Chem. Int. Ed.* **54**, 11162-11167 (2015).
8. Oka, K. *et al.* Poly(dihydroxybenzoquinone): its high-density and robust charge storage capability in rechargeable acidic polymer–air batteries. *Chem. Commun.* **56**, 4055-4058 (2020).
9. Oka, K., Murao, S., Kobayashi, K., Nishide, H. & Oyaizu, K. Charge- and Proton-Storage Capability of Naphthoquinone-Substituted Poly(allylamine) as Electrode-Active Material for Polymer–Air Secondary Batteries. *ACS Appl. Energy Mater.* **3**, 12019-12024 (2020).
10. Oka, K. *et al.* Conducting Redox Polymer as a Robust Organic Electrode-Active Material in Acidic Aqueous Electrolyte towards Polymer–Air Secondary Batteries. *ChemSusChem* **13**, 2280-2285 (2020).
11. Vo, T. K. *et al.* Facile Synthesis of UiO-66(Zr) Using a Microwave-Assisted Continuous Tubular Reactor and Its Application for Toluene Adsorption. *Cryst. Growth Des.* **19**, 4949-4956 (2019).
12. Sonal, S., Prakash, P., Mishra, B. K. & Nayak, G. C. Synthesis, characterization and sorption studies of a zirconium(iv) impregnated highly functionalized mesoporous activated carbons. *RSC Adv.* **10**, 13783-13798 (2020).
13. Li, X. *et al.* Baseline correction for infrared spectra using relative absorbance-based independent component analysis. *Opt. Express* **32**, 47137-47153 (2024).

14 Kozachuk, M. *et al.* Possible Radiation-Induced Damage to the Molecular Structure of Wooden Artifacts Due to Micro-Computed Tomography, Handheld X-Ray Fluorescence, and X-Ray Photoelectron Spectroscopic Techniques. *J. Conserv. Mus. Stud.* (2016).

15 Sánchez, F., Gutiérrez, M. & Douhal, A. Taking Advantage of a Luminescent ESIPT-Based Zr-MOF for Fluorochromic Detection of Multiple External Stimuli: Acid and Base Vapors, Mechanical Compression, and Temperature. *ACS Appl. Mater. Interfaces.* **15**, 56587-56599 (2023).

16 Mo, R.-J. *et al.* Regulating ion affinity and dehydration of metal-organic framework sub-nanochannels for high-precision ion separation. *Nat. Commun.* **15**, 2145 (2024).

17 Xu, X. *et al.* Visual and Gravimetric CO2 Sensing at High Humidity Levels Enabled by MOF-804 Cofunctionalized with Ionic Liquid and m-Cresol Purple. *Adv. Funct. Mater.* **35**, 2414141 (2025).

18 Åkerlund, L. *et al.* The Proton Trap Technology—Toward High Potential Quinone-Based Organic Energy Storage. *Adv. Energy Mater.* **7**, 1700259 (2017).

19 Steiner, T. The Hydrogen Bond in the Solid State. *Angew. Chem. Int. Ed.* **41**, 48-76 (2002).

20 Dong, D., Zhang, W., van Duin, A. C. T. & Bedrov, D. Grotthuss versus Vehicular Transport of Hydroxide in Anion-Exchange Membranes: Insight from Combined Reactive and Nonreactive Molecular Simulations. *J. Phys. Chem. Lett.* **9**, 825-829 (2018).

21 Ami, T., Oka, K., Kitajima, S. & Tohnai, N. Highly Fluorinated Nanospace in Porous Organic Salts with High Water Stability/Capability and Proton Conductivity. *Angew. Chem. Int. Ed.* **63**, e202407484 (2024).

22 Sadakiyo, M. & Kitagawa, H. Ion-conductive metal–organic frameworks. *Dalton Trans.* **50**, 5385-5397 (2021).

23 Liu, Y. *et al.* Highly Stable Metal–Organic Framework with Redox-Active Naphthalene Diimide Core as Cathode Material for Aqueous Zinc-Ion Batteries. *ChemSusChem* **16**, e202202305 (2023).

24 Deng, S., Xu, B., Zhao, J., Kan, C. W. & Liu, X. Unlocking Double Redox Reaction of Metal–Organic Framework for Aqueous Zinc-Ion Battery. *Angew. Chem. Int. Ed.* **63**, e202401996 (2024).

25 Nam, K. W. *et al.* Conductive 2D metal-organic framework for high-performance cathodes in aqueous rechargeable zinc batteries. *Nat. Commun.* **10**, 4948 (2019).

26 Sang, Z. *et al.* One-Dimensional π–d Conjugated Conductive Metal–Organic Framework with Dual Redox-Active Sites for High-Capacity and Durable Cathodes for Aqueous Zinc Batteries. *ACS Nano* **17**, 3077-3087 (2023).

27 Mondal, S., Samanta, P., Sahoo, R., Kuila, T. & Das, M. C. Porous and chemically robust MIL-100(V) MOF as an efficient cathode material for zinc-ion batteries. *Chem. Eng. J.* **470**, 144340 (2023).

28 Liu, J. *et al.* 2D Conductive Metal–Organic Framework with Anthraquinone Built-In Active Sites as Cathode for Aqueous Zinc Ion Battery. *Adv. Funct. Mater.* **34**, 2312636 (2024).

29 Li, J. *et al.* Interplanar space-controllable carboxylate pillared metal organic framework ultrathin nanosheet for superhigh capacity rechargeable alkaline battery. *Nano Energy* **62**, 876-882 (2019).

30 Li, Y. *et al.* Rechargeable Aqueous Polymer-Air Batteries Based on Polyanthraquinone Anode. *Chem* **5**, 2159-2170 (2019).

31 Choi, W., Harada, D., Oyaizu, K. & Nishide, H. Aqueous Electrochemistry of Poly(vinylanthraquinone) for Anode-Active Materials in High-Density and Rechargeable Polymer/Air Batteries. *J. Am. Chem. Soc.* **133**, 19839-19843 (2011).

32 Kawai, T., Oyaizu, K. & Nishide, H. High-Density and Robust Charge Storage with Poly(anthraquinone-substituted norbornene) for Organic Electrode-Active Materials in Polymer–Air Secondary Batteries. *Macromolecules* **48**, 2429-2434 (2015).

33 Oka, K., Murao, S., Kataoka, M., Nishide, H. & Oyaizu, K. Hydrophilic Anthraquinone-Substituted Polymer: Its Environmentally Friendly Preparation and Efficient Charge/Proton-Storage Capability for Polymer–Air Secondary Batteries. *Macromolecules* **54**, 4854-4859 (2021).

34 Zhong, L. *et al.* Redox Donor–Acceptor Conjugated Microporous Polymers as Ultralong-Lived Organic Anodes for Rechargeable Air Batteries. *Angew. Chem. Int. Ed.* **60**, 10164-10171 (2021).

35 Günther, T. *et al.* Redox-site accessibility of composites containing a 2D redox-active covalent organic framework: from optimization to application. *J. Mater. Chem. A* **11**, 13923-13931 (2023).

36 Ma, T. *et al.* Understanding the mechanism of a conjugated ladder polymer as a stable anode for acidic polymer-air batteries. *Joule* **7**, 2261-2273 (2023).

37 Tanaka, R. *et al.* Redox-Active Organic Molecules Supported by Polyhedral Oligomeric Silsesquioxane. *ChemNanoMat* **10**, e202400122 (2024).

38 Li, S., Hu, S., Li, H. & Han, C. Initiating a High-Rate and Stable Aqueous Air Battery by Using Organic N-Heterocycle Anode. *Angew. Chem. Int. Ed.* **63**, e202318885 (2024).
